# Supplementary material for: Sarcolemmal and mitochondrial membrane potentials measured ex vivo and in vivo in the heart by pharmacokinetic modelling of [99mTc]sestamibi
Source: J Physiol. 2026 Mar 16;604(7):2683–97. doi: 10.1113/JP290295 (PMC13039287; doi:10.1113/JP290295)
Supplement: Supplementary file 2 — Supplementary Information [file TJP-604-2683-s002.pdf]

Supplementary Information: Sarcolemmal and mitochondrial membrane potentials measured *ex vivo* and *in vivo* in the heart by pharmacokinetic modelling of [^{99m}Tc]sestamibi

Edward CT. Waters,[1] Friedrich Baark,[1] Matthew R. Orton,[2,3] Michael J. Shattock,[4,5] Richard Southworth,[1,5*] Thomas R. Eykyn[1,5*]

[1] School of Biomedical Engineering and Imaging Sciences, King's College London, The Rayne Institute, St. Thomas' Hospital, London, SE1 7EH, UK.

[2] Department of Radiology, MRI Unit, The Royal Marsden NHS Foundation Trust, London, UK.

[3] Division of Radiotherapy and Imaging, The Institute of Cancer Research, London, UK.

[4] School of Cardiovascular and Metabolic Medicine and Sciences, King's College London, United Kingdom.

[5] King's College London BHF Centre of Research Excellence

* Addresses for correspondence: Email: thomas.eykyn@kcl.ac.uk; richard.southworth@kcl.ac.uk

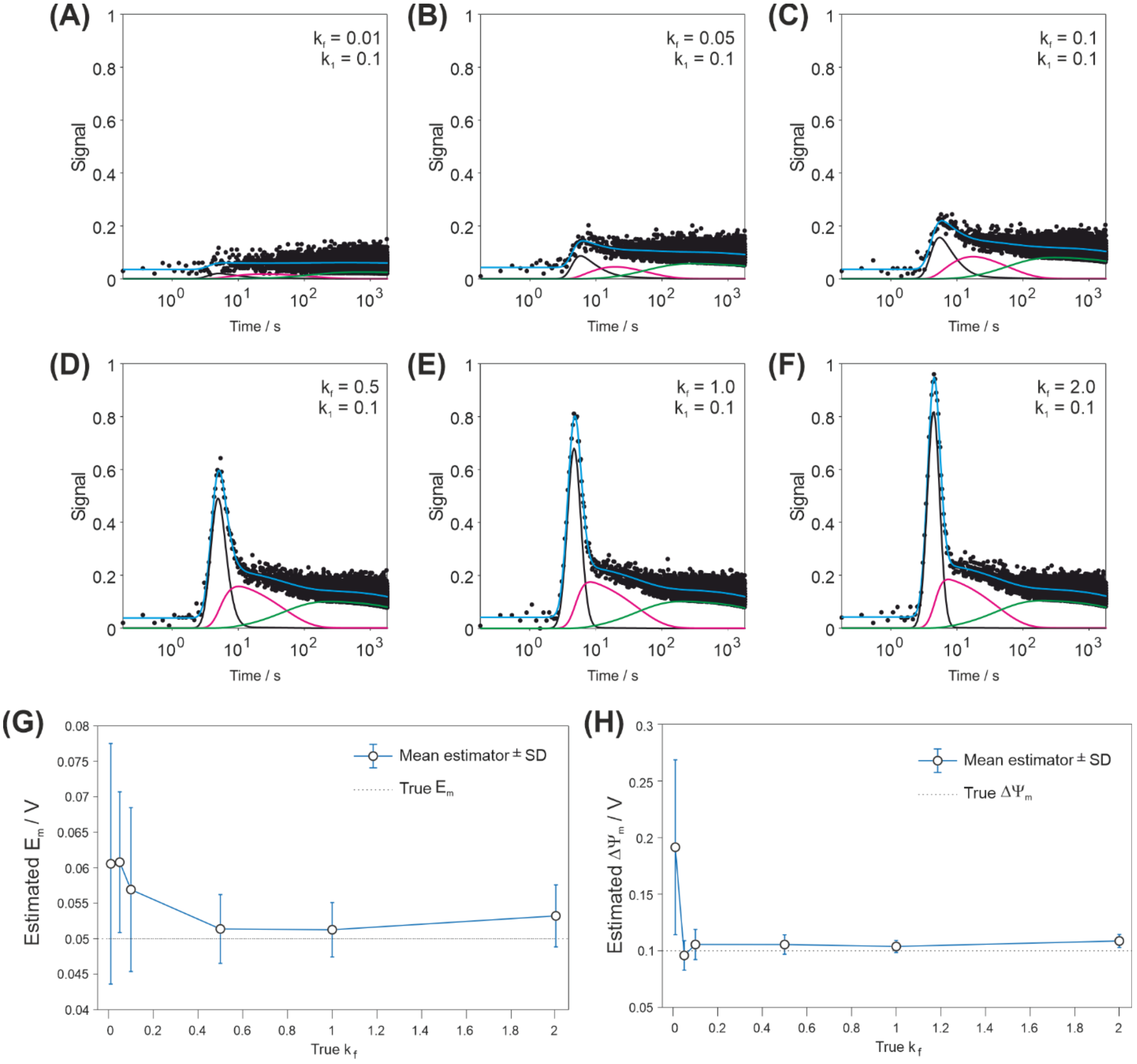

Figure S1. Simulated data exploring the influence of flow on estimation of membrane potentials. (A-F) Simulated time-activity curves as a function of the rate constant describing flow for k_f = (A) 0.01, (B) 0.05, (C) 0.1, (D) 0.5, (E) 1.0 and (F) 2.0 s^{-1}. The cyan line shows the best model fit; individual compartments are plasma ($c_p(t)$, black), cytosol ($c_c(t)$, magenta) and mitochondria ($c_m(t)$, green). MCMC fitting was performed with three trajectories with 10,000 iterations each and subsequently repeated 20 times to estimate mean ± SD for (G) sarcolemmal (E_m) and (H) mitochondrial ($\Delta\Psi_m$) membrane potentials from the fitting. The true values used for the simulations were $E_m = -50$ mV and $\Delta\Psi_m = -100$ mV dashed lines.

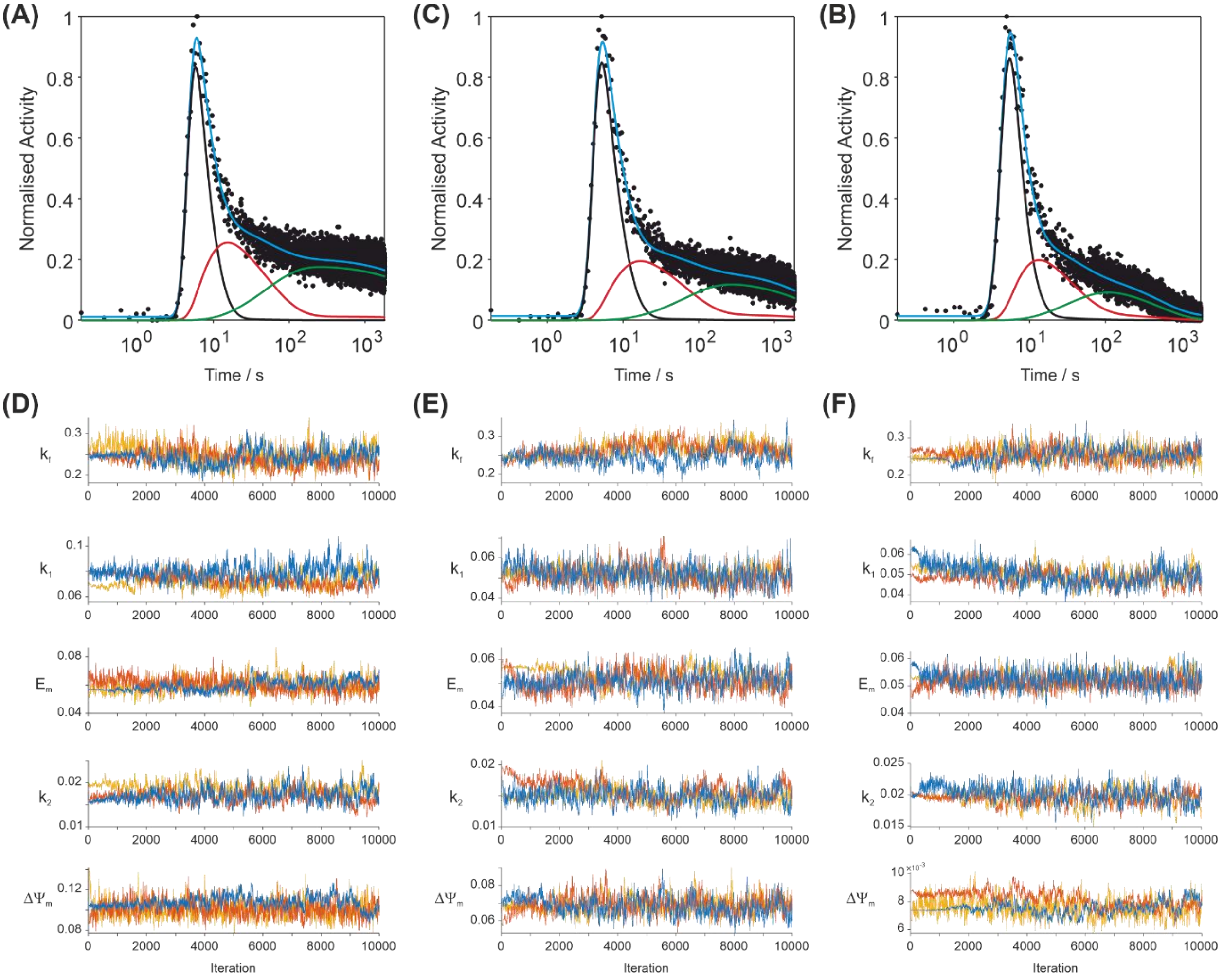

Figure S2. Experimental time-activity curves for [^{99m}Tc]tetrofosmin. Representative time activity curves following bolus injection of [^{99m}Tc]tetrofosmin into rat hearts perfused with (A) control Krebs–Henseleit buffer (KHB), (B) 300 nM CCCP and (C) 600 nM CCCP. The cyan line shows the best model fit; individual compartments are plasma ($c_p(t)$, black), cytosol ($c_c(t)$, magenta) and mitochondria ($c_m(t)$, green). (D-F) MCMC trajectories of the experimental data in (A-C) showing three independent trajectories (10,000 iterations each) for the rate constants k_f, k_1 and k_2 and the sarcolemmal (E_m) and mitochondrial ($\Delta\Psi_m$) membrane potentials.

Schematic summarizing steps in the fitting procedure

1. Load experimental data
• import time vector t and radioactive counts data
• time shift data, so bolus arrives at t_0 ~ 1 s
• remove negative time points
• normalize time-activity curve to peak activity

2. Differential equation model
• reparametrize rate constants k_{-1} and k_{-2} using Nernst eq.
• define voltages as positive fitting parameters
• define 3-compartment ODE system $dy(t)$
• define arterial input U(t) function
• solve for $y(t)$ using ode23s solver
• Ensure stability with safe ODE wrappers

3. LSQ initial parameter estimation
• initial guess θ_0 (jittered)
• weighted Poisson-like residuals
• minimize LSQ → parameter vector θ_{LSQ}
• compute Jacobian $J(\theta_{LSQ})$
• derive LSQ covariance and correlations

4. Transform parameters to φ-space
• log-transform parameters θ → φ – ensures positive
• don't transform delay t_0 - can be negative start time
• softmax-like transform for V_p, V_c, V_m
• result → parameter vector φ_{LSQ}

5. Define log-posterior in φ-space
• log p(φ | data) = loglike_Poisson(θ(φ)) + logPrior(φ)
• likelihood: Poisson count model
• prior: weak Gaussian centred at φ_{LSQ}
• automatically enforces positivity and volume bounds

6. Adaptive Metropolis MCMC sampling
• for each chain start at φ_{LSQ} + jitter
• proposal covariance adapts during burn-in
• accept/reject based on Metropolis-Hastings rule
• output → $\varphi_{samples}$ for each chain

7. Convert $\varphi_{samples} \rightarrow \theta_{samples}$ (physical params)
- exponentials for rates and amplitudes
- volume softmax inversion
- compile concatenated posterior samples

8. Posterior summaries & diagnostics
- posterior mean (Bayesian), median, standard deviation
- maximum a posteriori (MAP using max log-posterior)
- 95% credible intervals
- Trace plots, histograms
- Gelman–Rubin $\hat{R}$ for convergence

9. Plot fitted curves and compare
- LSQ fit vs Posterior Mean vs MAP
- Residual analysis
- Parameter correlations

10. Return final output structure
- θ_{LSQ}, covariance, correlations
- MCMC chains in φ and θ
- posterior stats (mean, median, MAP, CI)
- diagnostic metrics ($\hat{R}$)
- summary text for export

MATLAB fitting program

```matlab
function final = Tc_fitting_Bayes(fname)
% Tc_fitting_Bayes
% Single-file LSQ + Bayesian (Adaptive Metropolis) fitting
% for the 3-compartment model with arterial input.

    %% 1. Load and preprocess data
    [t,~,dataH,~] = textread(fname,'%f %f %f %f');
    data = dataH / max(dataH);

    t = t - 0;          % shift if needed
    data(t < 0) = [];
    t(t < 0)    = [];

    %% 2. LSQ initial fit
    % x = [ kf  k1   Es    k2   Em   t0  a   b A0 V1  V2  B    ]
    x0   = [ 0.5 0.1  0.05 0.01 0.1  1.1 12 4 1  0.3 0.3 0.01 ];
    x0   = x0 .* (1 + 0.02 * randn(1,12));  % jitter
    param_names = {'kf','k1','Es','k2','Em','t0','a','b','A0','V1','V2','B'};

    lb  = x0/5; ub  = 5*x0;
    lb(12) = 0.0; ub(12) = 0.1;
    lb(6) = 0; ub(6) = 2;

    opts = optimset('MaxIter',1e4,'MaxFunEvals',1e4,'TolFun',1e-4,'Display','iter');
    x_lsq = lsqnonlin(@(theta) poisson_residual(theta, t, data), x0, lb, ub, opts);
    % x_lsq = x0;
    phi_lsq = theta_to_phi_row(x_lsq);

    % --- Weak Gaussian priors in phi-space (centre at LSQ) ---
    mu_phi    = phi_lsq;

    sigma_phi = 0.1*ones(size(phi_lsq));

    % Refined call for Jacobian at solution
    optsJ = optimset('Display','off','MaxIter',0,'MaxFunEvals',200);
    [p_est,resnorm,residual,~,~,~,J] = lsqcurvefit(@Predict_invivo,x_lsq,t,data,[],[],optsJ);

    % Covariance from LSQ
    gamma = resnorm/(length(data) - length(p_est));
    H = full(J' * J);
    pCov = gamma * pinv(H);
    pSD   = sqrt(diag(pCov))';
    pCV   = 100 * pSD ./ abs(p_est);
    pCorr = pCov ./ sqrt(diag(pCov) * diag(pCov)');

    fprintf('\nLSQ parameters:\n');
    fprintf('  kf     k1     Es     k2     Em     t0    a     b     A0    V1    V2    B\n');
    fprintf(' %.3f  %.3f  %.3f  %.3f  %.3f  %.2f  %.2f  %.2f  %.2f  %.2f  %.2f  %.3f\n', x_lsq);
    fprintf('CV(%%):\n');
    disp(pCV);

    % Plot LSQ fit
    [M,M1,M2,M3] = Predict_invivo(x_lsq,t);
    figure;
    subplot(1,2,1);
    semilogx(t,data,'k.',t,M,'b',t,M1,'k',t,M2,'r',t,M3,'g','LineWidth',1);
    axis tight; axis square; xlim([1 max(t)]);
    xlabel('t'); ylabel('Signal');
    title('LSQ fit');

    subplot(1,2,2);
    semilogx(t,residual,'.k'); axis tight; axis square; xlim([min(t) max(t)]);
    xlabel('t'); ylabel('Residual');
    title('Residuals (LSQ)');
```

```
figure; imagesc(rot90(pCorr)); axis square;
title('Parameter correlation (LSQ)'); colorbar;


%%
function r = poisson_residual(theta, t, y)
    [M,~,~,~] = Predict_invivo(theta, t);
    y = y(:); M = M(:);
    w = sqrt(max(y, 1));
    r = (y - M) ./ w;
end


%% 3. Define log-posterior (Gaussian likelihood + box prior)
sigma2_est = var(residual);  % noise variance from LSQ residuals
logpost_fun = @(phi) logpost_phi_poisson(phi, data, t, mu_phi, sigma_phi);


%% 4. Adaptive Metropolis MCMC (multi-chain)
use_mcmc = input('Run Adaptive Metropolis MCMC? (y/n): ','s');
if any(lower(use_mcmc)=='y')
    nChains   = 3;
    nSamples  = input('Number of MCMC samples per chain (e.g. 20000): ');
    burn_in   = input('Burn-in samples per chain (e.g. 5000): ');
    thin      = input('Thinning factor (e.g. 5): ');

    % Initial covariance from LSQ
    % Simple diagonal proposal: small relative steps on each parameter
    step_frac = 1e-3;

    base_std = step_frac * max(abs(x_lsq), 1e-6);
    baseCov  = diag(base_std.^2);

    allChains = cell(nChains,1);

    % Work in phi-space
    for c = 1:nChains
        fprintf('\n=== Adaptive Metropolis chain %d/%d (phi-space) ===\n', c, nChains);

        % small jitter around LSQ in phi-space
        phiInit = phi_lsq .* (1 + 0.02*randn(size(phi_lsq)));

        % Simple diagonal proposal covariance in phi-space
        step_frac = 0.05;  % ~5% relative steps (tune if needed)
        base_std  = step_frac * ones(size(phi_lsq));
        baseCov   = diag(base_std.^2);

        [phi_chain, acc_rate] = adaptive_metropolis(logpost_fun, phiInit, baseCov, nSamples);
        fprintf('Chain %d acceptance rate: %.3f\n', c, acc_rate);
        allChains{c} = phi_chain;    % store chains in phi-space
    end

    % nChains        = numel(allChains);
    allChains_theta = cell(nChains,1);

    for c = 1:nChains
        phi_chain = allChains{c};              % [nSamples x 12] in phi
        nS        = size(phi_chain, 1);
        theta_chain = zeros(nS, 12);
        for i = 1:nS
            theta_chain(i,:) = phi_to_theta_row(phi_chain(i,:));
        end
        allChains_theta{c} = theta_chain;      % store [nSamples x 12] in theta
    end

    % Concatenate chains & thinning in phi-space
    idx_keep    = burn_in+1 : thin : nSamples;
    phi_keep_cell = cellfun(@(S) S(idx_keep,:), allChains, 'UniformOutput', false);
    phi_keep      = cell2mat(phi_keep_cell);    % [N_keep x 12] in phi-space
```

```matlab
    nKeep = size(phi_keep,1);
    theta_keep = zeros(nKeep, 12);
    for i = 1:nKeep
        theta_keep(i,:) = phi_to_theta_row(phi_keep(i,:));
    end

    % Posterior mean and MAP
    theta_mean   = mean(theta_keep, 1);
    theta_median = median(theta_keep, 1);
    theta_std    = std(theta_keep, 0, 1);

    % MAP: find phi with max logpost, then map to theta
    logpost_vals = arrayfun(@(i) logpost_fun(phi_keep(i,:)), 1:nKeep)';
    [~, iMAP]    = max(logpost_vals);
    phi_MAP      = phi_keep(iMAP,:);
    theta_MAP    = phi_to_theta_row(phi_MAP);

    % Build summary table as text
    summaryStr = sprintf('-----------------------------------------------------------------------\n');
    summaryStr = [summaryStr sprintf('\nPosterior summary from MCMC (after burn-in & thinning):\n') ];
    summaryStr = [summaryStr sprintf('Parameter  |  Mean  |  Median  |  SD  |  CV(%%)  |  MAP\n')   ];
    summaryStr = [summaryStr sprintf('-----------------------------------------------------------\n')];

    % Print header once
    fprintf('%s', summaryStr);

    for j = 1:numel(theta_mean)
        mu  = theta_mean(j);
        med = theta_median(j);
        sd  = theta_std(j);
        cv  = 100 * sd / abs(mu);
        map = theta_MAP(j);

       line = sprintf('%8s  | %10.4f  | %10.4f    | %10.4f  | %8.2f  | %10.4f\n', ...
               param_names{j}, mu, med, sd, cv, map);

       fprintf('%s', line);       % print to command window
       summaryStr = [summaryStr line];   % append to saved text
    end

    fprintf('\n');


    % Diagnostics
    diag_plots(allChains_theta);

    % Gelman-Rubin R-hat
    Rhat = gelman_rubin(allChains_theta, burn_in);
    fprintf('\nGelman-Rubin Rhat per parameter:\n');
    disp(Rhat);

    % Compare fits: LSQ vs posterior mean vs MAP
    compare_fits(t, data, x_lsq, theta_mean, theta_MAP);

    final.lsq            = x_lsq;
    final.pCov_lsq       = pCov;
    final.MCMC.allChains = allChains;
    final.MCMC.phi_samples   = phi_keep;     % transformed space
    final.MCMC.theta_samples = theta_keep;   % physical parameters
    final.MCMC.mean          = theta_mean;
    final.MCMC.median        = theta_median;
    final.MCMC.std           = theta_std;
    final.MCMC.MAP           = theta_MAP;
    final.MCMC.names     = param_names;
    final.MCMC.Rhat      = Rhat;
    final.MCMC.summary   = summaryStr;
else
    final.lsq      = x_lsq;
```

```
        final.pCov_lsq = pCov;
    end

end


%%
function logL = loglike_poisson(counts, t, theta)
    try
        [M, ~, ~, ~] = Predict_invivo(theta, t);
    catch
        logL = -Inf;
        return;
    end

    lambda = M(:);
    y      = counts(:);

    if numel(lambda) ~= numel(y)
        logL = -Inf;
        return;
    end

    % Clamp tiny/negative values to a small positive epsilon
    eps_lambda = 1e-6;
    if any(~isfinite(lambda))
        logL = -Inf;
        return;
    end
    lambda(lambda < eps_lambda) = eps_lambda;

    % Poisson log-likelihood (drop log(y!) constant term)
    logL = sum( y .* log(lambda) - lambda );
end


%% === Adaptive Metropolis =================================================
function [samples, acc_rate] = adaptive_metropolis(logpost, x0, C0, nSamples)
% Adaptive Metropolis (Haario et al. style)
% logpost: function handle, log-posterior
% x0:      initial parameter vector
% C0:      initial covariance matrix (approx)
% nSamples: number of samples

    d = numel(x0);
    samples = zeros(nSamples, d);
    samples(1,:) = x0;

    logp_curr = logpost(x0);
    acc = 0;

    % Tuning constants
    sd_scale = (2.4^2) / d;   % optimal scaling for Gaussian target
    eps_reg  = 1e-8;          % regularization

    % Make sure C0 is symmetric positive definite
    C0 = (C0 + C0')/2;
    C0 = C0 + 1e-6*eye(d);

    C  = C0;
    mu = x0;

    adapt_end = round(0.3 * nSamples);  % e.g. 30% burn-in for adaptation
    for i = 2:nSamples
        if i <= 50
            proposal_cov = C0;
        elseif i <= adapt_end
            proposal_cov = sd_scale * (C + eps_reg*eye(d));
            final_C = proposal_cov;  % remember last adapted cov
```

```
        else
            proposal_cov = final_C;  % fixed from here on
        end

        % Ensure symmetry
        proposal_cov = (proposal_cov + proposal_cov')/2;

        % Ensure positive definiteness using Cholesky + jitter
        jitter = 1e-8;
        maxTries = 6;
        success = false;
        for k = 1:maxTries
            [L,p] = chol(proposal_cov, 'lower');
            if p == 0
                success = true;
                break;
            else
                proposal_cov = proposal_cov + jitter*eye(d);
                jitter = jitter * 10;
            end
        end
        if ~success
            error('Proposal covariance not SPD even after jitter. Check your model / scaling.');
        end

        % Draw Gaussian jump: N(0, proposal_cov)
        jump = randn(1,d) * L';   % 1-by-d

        x_prop = samples(i-1,:) + jump;

        % Evaluate log-posterior at proposed point
        logp_prop = logpost(x_prop);
        if ~isfinite(logp_prop)
            samples(i,:) = samples(i-1,:);
            continue;
        end

        % Metropolis–Hastings acceptance
        alpha = logp_prop - logp_curr;
        if log(rand) < alpha
            samples(i,:) = x_prop;
            logp_curr = logp_prop;
            acc = acc + 1;
        else
            samples(i,:) = samples(i-1,:);
        end

        % Online update of mean and covariance
        x = samples(i,:);
        delta = x - mu;
        mu = mu + delta / i;
        C = (i-2)/(i-1) * C + (delta'*(x - mu)) / i;
    end

    acc_rate = acc/(nSamples-1);
end


%% === Gradient of log-posterior (finite differences) =====================
function g = grad_logposterior_fd(logpost, theta)
    d = numel(theta);
    g = zeros(1,d);
    eps = 1e-4 * (1 + abs(theta));

    f0 = logpost(theta);
    for j = 1:d
        e = zeros(1,d);
        e(j) = eps(j);
        fp = logpost(theta + e);
```

```
        fm = logpost(theta - e);
        g(j) = (fp - fm) / (2*eps(j));
    end
end


%% === Diagnostics: traces, histograms, R-hat =============================
function diag_plots(allChains)
    nChains = numel(allChains);

    % Make sure all chains are matrices and find common length
    lengths = zeros(nChains,1);
    for c = 1:nChains
        S = allChains{c};
        if isempty(S)
            error('Chain %d is empty.', c);
        end
        lengths(c) = size(S,1);
    end
    nSamp = min(lengths);
    d     = size(allChains{1},2);

    % Truncate all chains to common length
    for c = 1:nChains
        allChains{c} = allChains{c}(1:nSamp,:);
    end

    nShow = min(d, 6);

    % --- Trace plots ---
    figure;
    for j = 1:nShow
        subplot(nShow,1,j); hold on;
        for c = 1:nChains
            plot(allChains{c}(:,j));
        end
        hold off;
        ylabel(sprintf('\\theta_%d',j));
        if j==1, title('Trace plots (first parameters)'); end
    end
    xlabel('Iteration');

    % --- Histograms ---
    figure;
    for j = 1:nShow
        subplot(2,ceil(nShow/2),j); hold on;
        for c = 1:nChains
            S = allChains{c}(:,j);
            S = S(isfinite(S));
            if ~isempty(S)
                histogram(S,20,'Normalization','pdf');
            end
        end
        hold off;
        xlabel(sprintf('\\theta_%d',j));
        if j==1, title('Marginal posteriors (first parameters)'); end
    end
end


%%
function Rhat = gelman_rubin(allChains, burn_in)
% allChains: cell, each [nSamples x d]
% burn_in:   integer

    nChains = numel(allChains);
    [nSamples, d] = size(allChains{1});

    idx = burn_in+1 : nSamples;
```

```matlab
    m = numel(idx);

    chain_means = zeros(nChains, d);
    chain_vars  = zeros(nChains, d);

    for c = 1:nChains
        S = allChains{c}(idx,:);
        chain_means(c,:) = mean(S,1);
        chain_vars(c,:)  = var(S,0,1);
    end

    grand_mean = mean(chain_means,1); %#ok<NASGU>

    % Between-chain variance
    B = m * var(chain_means,0,1);

    % Within-chain variance
    W = mean(chain_vars,1);

    % Estimate of marginal posterior variance
    var_hat = ( (m-1)/m ) .* W + (1/m) .* B;

    Rhat = sqrt(var_hat ./ W);
end


%% === Comparison of LSQ vs posterior mean vs MAP =========================
% Least square vs Posterior Mean (Bayesian mean estimate) vs MAP (Maximum A Posteriori)
function compare_fits(t, data, theta_lsq, theta_mean, theta_MAP)

    tPlot    = t(:);
    dataPlot = data(:);

    [M_lsq,~,~,~]  = Predict_invivo(theta_lsq, tPlot);
    [M_mean,~,~,~] = Predict_invivo(theta_mean, tPlot);
    [M_map,~,~,~]  = Predict_invivo(theta_MAP, tPlot);

    M_lsq  = M_lsq(:);
    M_mean = M_mean(:);
    M_map  = M_map(:);

    % Only positive finite times and finite model values
    mask = tPlot > 0 & isfinite(dataPlot) & ...
           isfinite(M_lsq) & isfinite(M_mean) & isfinite(M_map);

    tPlot    = tPlot(mask);
    dataPlot = dataPlot(mask);
    M_lsq    = M_lsq(mask);
    M_mean   = M_mean(mask);
    M_map    = M_map(mask);

    figure;
    semilogx(tPlot,dataPlot,'k.', ...
             tPlot,M_lsq,'b-', ...
             tPlot,M_mean,'r--', ...
             tPlot,M_map,'g-.','LineWidth',1.2);
    legend('Data','LSQ','Posterior mean','MAP','Location','Best');
    xlabel('t'); ylabel('Signal');
    axis tight; axis square;
    if ~isempty(tPlot)
        xlim([min(tPlot) max(tPlot)]);
    end
    title('LSQ vs Posterior mean vs MAP');
end


%%
function lp = logpost_box_poisson(theta, counts, t, lb, ub)
    % Hard box prior: theta must lie in [lb, ub]
```

```
    if any(theta < lb) || any(theta > ub)
        lp = -Inf;
        return;
    end

    % Inside bounds: use Poisson likelihood
    lp = loglike_poisson(counts, t, theta);
end

%%
function lp = logpost_phi_poisson(phi, counts, t, mu_phi, sigma_phi)
    theta = phi_to_theta_row(phi);

    if any(~isfinite(theta))
        lp = -Inf;
        return;
    end

    % Poisson likelihood in theta-space
    logL = loglike_poisson(counts, t, theta);
    if ~isfinite(logL)
        lp = -Inf;
        return;
    end

    % Weak Gaussian priors in phi-space
    z = (phi - mu_phi) ./ sigma_phi;
    logPrior = -0.5 * sum(z.^2);

    lp = logL + logPrior;
end



%% === Forward model: Predict_invivo, ODE, arterial input =================
function [M, M1, M2, M3] = Predict_invivo(xIn, tIn)
    % Solve at requested time points
    % odeOpts = odeset('RelTol',1e-4, 'AbsTol',1e-8);  % adjust if needed

    [tSol, y] = ode15s(@rigid_invivo, tIn, [0,0,0], [], xIn);

    % If solver didn’t return all points, treat as failure
    if numel(tSol) ~= numel(tIn)
        error('ODE23s failed to evaluate solution at all requested time points');
    end

    V1  = xIn(10);
    V2  = xIn(11);
    V3  = 1 - V1 - V2;
    B   = xIn(12);

    M1 = V1 * y(:,1);
    M2 = V2 * y(:,2);
    M3 = V3 * y(:,3);
    M  = M1 + M2 + M3 + B;
end


%%
function dy = rigid_invivo(t, y, xIn)
    dy = zeros(3, 1);
    R = 8.314;
    T = 310;
    F = 96485;

    kf  = xIn(1);
    k1  = xIn(2);
    k_1 = xIn(2) * exp((-xIn(3) * F) / (R * T));
    k2  = xIn(4);
```

```
    k_2 = xIn(4) * exp((-xIn(5) * F) / (R * T));

    kloss = 3.2059e-05;
    U = ArterialInput(t - xIn(6), xIn(7), xIn(8), xIn(9));

    dy(1) =  kf * U - (kf + k1 + kloss) * y(1) + k_1 * y(2);
    dy(2) =  k1 * y(1) - (k_1 + k2 + kloss) * y(2) + k_2 * y(3);
    dy(3) =  k2 * y(2) - (k_2 + kloss) * y(3);
end


%%
function c = ArterialInput(tH, a, b, A0)
    c = zeros(size(tH));
    idx = tH >= 0;
    tPos = tH(idx);
    c(idx) = A0 * (tPos.^a) .* exp(-tPos * b);
end


%%
function theta = phi_to_theta_row(phi)

    % Rates (positive)
    kf = exp(phi(1));
    k1 = exp(phi(2));
    Es = exp(phi(3));
    k2 = exp(phi(4));
    Em = exp(phi(5));

    % Delay (free, can be negative)
    t0 = phi(6);

    % AIF shape (positive)
    a  = exp(phi(7));
    b  = exp(phi(8));
    A0 = exp(phi(9));

    % Volume fractions V1, V2, V3 = 1 - V1 - V2
    e1 = exp(phi(10));
    e2 = exp(phi(11));
    e3 = 1;
    Z  = e1 + e2 + e3;

    V1 = e1 / Z;
    V2 = e2 / Z;
    % V3 = 1 - V1 - V2   (inside Predict_invivo)

    % Background (positive)
    B = exp(phi(12));

    theta = [kf, k1, Es, k2, Em, t0, a, b, A0, V1, V2, B];
end


%%
function phi = theta_to_phi_row(theta)
    % theta = [kf k1 Es k2 Em t0 a b A0 V1 V2 B]

    kf = theta(1);
    k1 = theta(2);
    Es = theta(3);
    k2 = theta(4);
    Em = theta(5);
    t0 = theta(6);
    a  = theta(7);
    b  = theta(8);
    A0 = theta(9);
    V1 = theta(10);
```

```
    V2 = theta(11);
    B  = theta(12);

    V3 = max(1 - V1 - V2, 1e-6);
    e1 = V1 / V3;
    e2 = V2 / V3;
    z1 = log(max(e1, 1e-12));
    z2 = log(max(e2, 1e-12));

    phi = zeros(1,12);
    phi(1)  = log(max(kf, 1e-12));
    phi(2)  = log(max(k1, 1e-12));
    phi(3)  = log(max(Es, 1e-12));
    phi(4)  = log(max(k2, 1e-12));
    phi(5)  = log(max(Em, 1e-12));
    phi(6)  = t0;
    phi(7)  = log(max(a,  1e-12));
    phi(8)  = log(max(b,  1e-12));
    phi(9)  = log(max(A0, 1e-12));
    phi(10) = z1;
    phi(11) = z2;
    phi(12) = log(max(B,  1e-12));
end
```
